# The overlooked role of primary care in early childhood trauma: comment on ‘Early childhood trauma and its long-term impact on cognitive and emotional development’

**DOI:** 10.1080/07853890.2026.2635597

**Published:** 2026-02-27

**Authors:** Erhan Şimşek, Öykü Su Tulumtaş

**Affiliations:** ^a^ Department of Family Medicine, Ankara Yıldırım Beyazıt University Faculty of Medicine, Ankara, Türkiye; ^b^ Ministry of Health of the Republic of Türkiye, Directorate General of Public Health, Ankara, Türkiye

Dear Editor

We read with great interest the article by Fan and Kang entitled ‘Early childhood trauma and its long-term impact on cognitive and emotional development: a systematic review and meta-analysis,’ published in Annals of Medicine [1]. The authors address a highly relevant and timely topic, and the manuscript provides a valuable synthesis of the long-term cognitive and emotional consequences of early childhood trauma.

While the review comprehensively examines neurocognitive and emotional outcomes, we would like to highlight an important perspective that appears to be underrepresented: the role of family medicine and primary care in the early recognition and prevention of childhood trauma and abuse.

Family physicians are often the first and most continuous point of contact for children and their families. Through routine well-child visits, vaccination follow-ups, and longitudinal care, primary care clinicians are uniquely positioned to identify early signs of neglect, abuse, and adverse childhood experiences (ACEs). Subtle behavioural changes, developmental delays, recurrent somatic complaints, and psychosocial risk factors within the family context can often be recognised early in primary care settings [2].

Recent evidence indicates that structured ACE-related training, screening, and response models implemented in primary care improve clinicians’ ability to identify childhood adversity and to initiate appropriate clinical and psychosocial interventions [3]. Furthermore, systematic reviews suggest that ACE screening in primary care is feasible and increasingly adopted, supporting early engagement with affected children and families and facilitating timely referral and support pathways [4].

Beyond early detection, family medicine plays a critical role in prevention. Preventive counselling, parental education, anticipatory guidance, and early psychosocial interventions are core components of family practice and have been associated with resilience-building and mitigation of the long-term health consequences of childhood trauma [4,5]. In this context, trauma-informed primary care approaches represent an essential element of comprehensive, community-oriented health systems by strengthening continuity of care and promoting early preventive action [3–5].

The absence of an explicit discussion of family medicine and primary care in the reviewed article represents a missed opportunity to emphasise a key preventive and multidisciplinary dimension of early childhood trauma. Incorporating this perspective would further strengthen the manuscript by providing a more holistic view of prevention strategies and underscoring the importance of integrating primary care into efforts to reduce the long-term impact of early childhood trauma.

We hope that this perspective will be considered in future discussions and publications addressing early childhood trauma and abuse.

Sincerely,

Erhan Şimşek, MD, Assistant Professor

Öykü Su Tulumtaş, MD, Public Health Specialist

## Data Availability

Data availability does not apply to this article as no new data were created or analysed in this study.
